# Supplementary material for: Biomarkers of Neutrophil Activation in Patients with Symptomatic Chronic Peripheral Artery Disease Predict Worse Cardiovascular Outcome
Source: Biomedicines. 2023 Mar 12;11(3):866. doi: 10.3390/biomedicines11030866 (PMC10045814; doi:10.3390/biomedicines11030866)
Supplement: Supplementary file 1 [file biomedicines-11-00866-s001.zip › biomedicines-2251090-supplementary material - corrected.pdf]

1. Supplementary Methods

1.1. Vascular assessment in patients with PAD

1.1.1. Limb perfusion: ABI and TBI calculation, TcPO2 measurement

The ankle–brachial index (ABI) calculation was performed in supine patients following the guidelines of the European Society of Cardiology (ESC) and the European Society for Vascular Surgery (ESVS) [1], as well as those of the European Society of Vascular Medicine (ESVM) [2], using a vascular handheld 4-8 MHz Doppler instrument (Basic-2, Atys Medical, 69510 Soucieu en Jarrest, France). ABI was calculated separately for each leg, dividing the higher of the posterior tibialis or dorsalis pedis systolic blood pressure (SBP) by the higher of the right or left arm SBP. In the case of abnormally high ABI (i.e., ABI > 1.4), the systolic toe pressure was then measured (Basic-2, Atys Medical, 69510 Soucieu en Jarrest, France), and the toe–brachial index (TBI) was calculated. The test was considered diagnostic for PAD if the TBI was < 0.7 [1]. ABI and TBI values of the most-affected leg were considered.

Transcutaneous oximetry (TcPO2) (mmHg) was measured with the patient in a supine position. Electrodes were positioned at the dorsum of the foot (on distal and proximal foot) following a stabilization period of 15 minutes using a TCM4 Radiometer (Medical Aps). TcPO2 values of the most-affected leg were considered.

1.1.2 Walking functional capacity: constant-load treadmill and 6-minute walking tests

Patients underwent a constant-load treadmill test at 3.2 km/h speed with a 12% slope [3] to determine pain-free walking distance (PFWD) (m) and maximal walking distance (MWD) (m). Speed was adapted depending on patients' safety and exercise tolerance. Following the treadmill test, patients were asked to immediately lie down in a supine position. The post-exercise ankle SBP (mmHg) was assessed, and the post-exercise ABI decrease (%) was calculated. The post-exercise ABI decrease of the most-affected leg was considered.

Patients were asked to walk as far as possible within 6 min in an indoor 50 m corridor to determine the 6-minute pain-free walking distance (6PFWD) (m) and maximal walking distance (6MWD) (m). Patients were allowed to stop during the test. While resting, they were allowed to lean against the wall, but they were instructed to resume walking as soon as they could, according to guidelines [4].

1.1.3. Arterial stiffness: carotid–femoral pulse wave velocity

After at least 10 minutes at rest, right-side carotid–femoral pulse wave velocity (PWV) was measured in triplicate with Complior Analyse (ALAM medical, France) under resting conditions following a standardized the protocol with bilateral blood pressure measurement, taking the higher value as reference for the PWV, measuring the carotid–femoral distance and automatic correction to 80% according to guidelines [5]. The carotid pulse was captured with the sensor fixed in a clamp or manually, and the femoral pulse was captured manually. The PWV was calculated with the Complior Analyse by calculating the crossing of the two tangents marking the beginning of the pulse wave. Only results reaching quality levels above 93% were accepted. Mean values were calculated.

1.1.4. Endothelial function: flow-mediated dilation of the brachial artery

Subjects were prepared for flow-mediated dilatation (FMD) as follows: fasting 8 hours before testing, no tobacco, caffeine or cocoa consumption during the last 12 h. Vitamin C and E preparations and anti-inflammatory drugs were stopped 72 h before the tests. Patients were instructed to avoid strenuous physical activity before testing.

All tests were conducted by one board-certified vascular physician and ultrasound specialist.

After 20 minutes at rest, at room temperature, endothelial function was measured according to current guidelines [6] with either an ALOKA Prosound Alpha 10 or Prosound F75 ultrasound machine (Hitachi Aloka medical Ltd., Japan), which both calculate the FMD semi-automatically, using continuous automatic arterial wall detection by radiofrequency during the whole test from baseline until 5 minutes after, and included manual control of FMD peak detection. During the whole test, electrocardiogram monitoring was mandatory.

The patient's right arm was immobilized in a custom-made splint with a gap for the blood pressure cuff to avoid unwanted movements during cuff inflation. The pulsed wave Doppler sample volume included the whole artery, and was measured with a maximum angle of 60°, allowing correct flow volume and velocity measurement at baseline and after ischemic challenge.

A linear 10 MHZ ultrasound transducer was fixed above patient's right elbow with a probe holder, searching an optimal entered long axis view of the brachial artery with good visualization of the double lines of Pignoli (intima–media thickness). A blood pressure cuff was fixed on the forearm.

First, baseline brachial artery diameter was measured in the upper arm for at least 60 seconds. If the baseline was stable, we proceeded to ischemic testing and inflated the cuff until 50 mmHg above systolic blood pressure for 5 minutes. During the whole duration ischemic challenge, patients' tolerance was observed.

If the baseline was unstable, we repeated the procedure. After cuff deflation, flow velocity and flow volume were measured for 15 seconds, and artery diameter increase was analyzed semi-automatically for 5 minutes. FMD was automatically calculated, always with visual assessment of correct FMD peak positioning.

2. Supplementary Tables

Table S1. Linear correlation analyses for markers of neutrophil activation and PAD severity.

	**PMN-elastase (ng/mL)**	**NGAL (ng/mL)**	**MPO (ng/mL)**	**MPO-DNA (abs 405-490)**
**ABI**	0.058 (0.662)	−0.094 (0.481)	0.054 (0.689)	0.019 (0.885)
**TBI**	−0.162 (0.401)	−0.176 (0.362)	0.129 (0.514)	0.187 (0.342)
**TcPO2** (mmHg)				
Distal foot	−0.066 (0.617)	0.043 (0.745)	0.061 (0.644)	−0.206 (0.118)
Proximal foot	−0.170 (0.210)	−0.081 (0.540)	−0.009 (0.946)	−0.284 (0.029)
**FMD**				
Diastole (%)	−0.115 (0.372)	−0.246 (0.054)	−0.180 (0.166)	−0.221 (0.087)
Velocity (%)	−0.076 (0.561)	0.040 (0.761)	−0.104 (0.434)	0.173 (0.190)
Flow volume (%)	−0.137 (0.295)	−0.127 (0.335)	−0.013 (0.920)	0.006 (0.964)
Time to peak (s)	−0.068 (0.601)	0.097 (0.451)	0.067 (0.614)	0.002 (0.985)
**PWV** (m/s)	−0.091 (0.498)	−0.157 (0.240)	0.038 (0.772)	0.036 (0.789)
**6MWT** (m)				
6PFWD	-0.141 (0.296)	−0.298 (0.025)	−0.154 (0.257)	0.004 (0.974)
6MWD	-0.023 (0.866)	−0.243 (0.066)	−0.056 (0.679)	0.015 (0.912)
**Treadmill test**				
PFWD (m)	-0.034 (0.795)	−0.256 (0.046)	−0.006 (0.962)	−0.120 (0.363)
MWD (m)	0.048 (0.715)	−0.305 (0.017)	−0.040 (0.762)	−0.153 (0.244)
Post-exercise ABI decrease (%)	0.192 (0.138)	−0.060 (0.644)	0.151 (0.250)	0.026 (0.845)

Results are expressed as ϱ (*p*-value). *p*-values are in bold if statistically significant. ABI: ankle–brachial index; FMD: flow-mediated dilation; MPO: myeloperoxidase; NGAL: neutrophil gelatinase-associated lipocalin; PMN: polymorphonuclear neutrophil; PWV: pulse wave velocity; TBI: toe–brachial index; TcPO2: transcutaneous oxygen pressure; 6MWT: 6-minute walking test; 6MWD: 6-minute maximal walking distance; 6PFWD: 6-minute pain-free walking distance.

Table S2. PAD patients' characteristics at baseline according to MACE and/or MALE at 6-month follow-up.

		MACE and/or MALE		
	**All subjects (= 63)**	**No Outcome (= 52)**	**Outcome (= 11)**	***p***
**Clinical characteristics**				
Sex (male) (%)	44 (69.8)	34 (65.4)	10 (90.9)	0.089
Age (years) (± SD)	64.0 (9.0)	64.8 (9.0)	60.5 (8.4)	0.121
BMI (kg/m2) (± SD)	26.8 (5.2)	27.2 (5.3)	25.0 (4.4)	0.180
Waist (cm) (± SD)	99.7 (16.1)	101.2 (17.2)	93.8 (8.9)	0.183
**Leriche–Fontaine PAD stage**				
Stage IIb or more (%)	14 (22.2)	10 (19.2)	4 (36.4)	0.399
**Comorbidities**				
Obesity (%)	12 (19.0)	11 (21.2)	1 (9.1)	0.326
Diabetes (%)	22 (34.9)	19 (36.5)	3 (27.3)	0.416
Active smoking (%)	33 (52.4)	25 (48.1)	8 (72.7)	0.124
Hypertension (%)	43 (68.3)	38 (73.1)	5 (45.5)	0.079
CAD history (%)	19 (30.2)	15 (28.8)	4 (36.4)	0.435
CeVD history (%)	14 (22.2)	13 (25.0)	1 (9.1)	0.234
VTE history (%)	7 (11.1)	6 (11.5)	1 (9.1)	0.647
Previous lower limb revasculari-zation (%)	33 (52.4)	27 (51.9)	6 (54.5)	0.570
**Treatment**				
Aspirin (%)	48 (76.2)	38 (73.1)	10 (90.9)	0.272
Statin (%)	54 (86.7)	46 (88.5)	8 (72.7)	0.184
ACEi/ARB (%)	37 (59.7)	31 (60.8)	6 (54.5)	0.744
**Laboratory**				
Total cholesterol (md/dL) (± SD)	166.7 (32.3)	167.2 (31.9)	164.3 (35.5)	0.810
LDL cholesterol (mg/dL) (± SD)	87.3 (29.0)	87.5 (28.5)	86.2 (33.0)	0.912
HDL cholesterol (mg/dL) (± SD)	50.2 (18.4)	51.8 (19.5)	42.9 (10.3)	0.170
Triglycerides (mg/dL) (± SD)	153.2 (91.6)	148.2 (86.1)	175.2 (115.3)	0.681

ACEi: angiotensin-converting enzyme inhibitor; ARB: angiotensin receptor blocker; BMI: body mass index; CAD: coronary artery disease; CeVD: cerebrovascular disease; HDL: high-density lipoprotein; LDL: low-density lipoprotein; MACE: major adverse cardiovascular event; MALE: major adverse limb event; SD: standard deviation; VTE: venous thromboembolism.

References

1. Aboyans, V.; Ricco, J.-B.; Bartelink, M.-L.E.L.; Björck, M.; Brodmann, M.; Cohnert, T.; Collet, J.-P.; Czerny, M.; De Carlo, M.; Debus, S.; et al. 2017 ESC Guidelines on the Diagnosis and Treatment of Peripheral Arterial Diseases, in collaboration with the European Society for Vascular Surgery (ESVS). *Eur. Heart J.* **2018**, *39*, 763–816, doi:10.1093/eurheartj/ehx095.
2. Frank, U.; Nikol, S.; Belch, J.; Boc, V.; Brodmann, M.; Carpentier, P.H.; Chraim, A.; Canning, C.; Dimakakos, E.; Gottsäter, A.; et al. ESVM Guideline on peripheral arterial disease. *Vasa* **2019**, *48*, 1–79, https://doi.org/10.1024/0301-1526/a000834.
3. Treat-Jacobson, D.; McDermott, M.M.; Bronas, U.G.; Campia, U.; Collins, T.C.; Criqui, M.H.; Gardner, A.W.; Hiatt, W.R.; Regensteiner, J.G.; Rich, K.; et al. Optimal Exercise Programs for Patients With Peripheral Artery Disease: A Scientific Statement From the American Heart Association. *Circulation* **2019**, *139*, e10–e33, https://doi.org/10.1161/cir.0000000000000623.
4. ATS Committee on Proficiency Standards for Clinical Pulmonary Function Laboratories. American Thoracic Society. ATS statement: Guidelines for the six-minute walk test. *Am. J. Respir. Crit. Care Med.* **2002**, *166*, 111–117, doi:10.1164/ajrccm.166.1.at1102.
5. Van Bortel, L.M.; Laurent, S.; Boutouyrie, P.; Chowienczyk, P.; Cruickshank, J.; De Backer, T.; Filipovsky, J.; Huybrechts, S.; Mattace-Raso, F.U.; Protogerou, A.; et al. Expert consensus document on the measurement of aortic stiffness in daily practice using carotid-femoral pulse wave velocity. *J. Hypertens.* **2012**, *30*, 445–448, https://doi.org/10.1097/hjh.0b013e32834fa8b0.
6. Thijssen, D.H.J.; Bruno, R.M.; Van Mil, A.C.C.M.; Holder, S.M.; Faita, F.; Greyling, A.; Zock, P.L.; Taddei, S.; Deanfield, J.E.; Luscher, T.; et al. Expert consensus and evidence-based recommendations for the assessment of flow-mediated dilation in humans. *Eur. Hear. J.* **2019**, *40*, 2534–2547, https://doi.org/10.1093/eurheartj/ehz350.
